# Supplementary material for: Distributions of extinction times from fossil ages and tree topologies: the example of mid-Permian synapsid extinctions
Source: PeerJ. 2021 Dec 9;9:e12577. doi: 10.7717/peerj.12577 (PMC8667717; doi:10.7717/peerj.12577)
Supplement: Supplemental Information 1 — Archive of the github repository [file peerj-09-12577-s001.zip › DateFBD-master/User_Manual_rfbd.pdf]

User manual
Package rfbd

Introduction

The package rfbd implements the exact computation of the distribution of the divergence and the extinction times of a tree topology or a set of tree topologies, of extant and/or extinct taxa, including fossils, in which the only temporal information is given by the fossil ages and the origin time of diversification, undre the Fossilized Birth Death (FBD) model. The fossil ages can be provided as (stratigraphic) intervals of time, on which we integrate the distributions computed. We also have to integrate among the possible speciation, exinction and fossilization rates. In both case, the integration is performed by using an importance sampling procedure. The biased density used in this importance sampling procedure weights each combination of possible fossil ages and rates proportionally to the probability of the tree with these fossil ages under the fossilized birth and death model with these rates. It allows us to obtain posterior densities for the speciation, exinction and fossilization rates, (and the fossil ages).

Installation

Using the package rfbd first requires to install it in your R system by typing

```
>install.packages("<path_to>/rfbd_0.1.tar.gz", repos = NULL, type = "source")
```

in a R console, where `path_to` is the path to the file '`rfbd_0.0.tar.gz`'.

The package can then be loaded anytime with

```
>library(rfbd)
```

Using the package rfbd

For technical reasons, all the functionalities of the standalone software are not implemented in the package rfbd yet. In particular, the standalone software includes graphical features which are not implemented in the the package rfbd since they require an external library. More importantly, the refbd software does not use multithreading (paralell computations) since the library required by the standalone software is not available on all the operating systems. This point limits the ability of the package rfbd to deal with big dataset with regard to the standalone software. Notably, the Cotylasauria dataset containing the 1000 equiparsimonious trees is too lage to be handled by the R package (computations tooks several hours with 40 threads working in paralell). We provide a samller dataset containing only the tree of dimetrodons '`Dimetrodon.phy`' and a list of taxa deining a sub-clade of this tree '`Clade_Dimetrodon.txt`' to be used with the package rfbd.

The package rfbd includes four functions `FBDdiagnostic`, `FBDspeciation_distribution`, `FBDextinction_distribution` and `FBDextinction_upperBound`. All the functions have numerous parameters, mainly to control the proposal of MCMC (cf their R manual). `FBDdiagnostic` does not have a return value and just writes two coda files for MCMC diagnostic purposes (all the other functions also write the coda files).

Assuming that all the files are in the R working directory, one calls `FBDdiagnostic` with

```
>FBDdiagnostic(treeName="Dimetrodon.phy", fossilName="CotylosauriaAges.csv", outName = "Dimetrodon.
out", indName = "Dimetrodon.ind", inf_origin=-400, sup_origin=NaN, end_time=0., spec_wind_size=0.1,
```

```
exti_wind_size=0.1, foss_wind_size=0.02, spec_init=0.5, exti_init=0.5, foss_init=0.1, iter=1000,
prop = 0.75, al = 0.75, burn = 25000, thin = 200, step = 0.01)
```

The two files `Dimetrodon.out` and `Dimetrodon.ind` produces by the command just above can then be read for instance by using the R package coda with

```
>library(coda)

>MCMC_samples = read.coda("Dimetrodon.out", "Dimetrodon.ind")
```

Various MCMC diagnostics can then be performed (see `https://cran.r-project.org/web/packages/coda/coda.pdf`).

If one is interested by the posterior distribution of the parameters of the model. In particular, their Highest Posterior Density intervals can be computed by the function `HPDinterval` of the coda package, i.e., with the command line :

```
>HPDinterval(MCMC_samples, 0.95)
```

Plotting the histogram of the posterior distribution of a parameter of the model, e.g., the speciation rate (Fig. 1-left), can be performed by

```
>hist(as.matrix(MCMC_samples)[,"birth"], xlab="speciation rate", breaks=40)
```

`FBDspeciation_distribution` returns the distribution or the density of the divergence times associated to the root of the smallest clade containg all the taxa in the first parameter (which should be a clade for all the trees if several are provided) and still writes two coda files for diagnostic purposes.

```
>distSpe = FBDspeciation_distribution("Clade_Dimetrodon.txt", treeName="Dimetrodon.phy",
fossilName="CotylosauriaAges.csv", outName = "Dimetrodon.out", indName = "Dimetrodon.ind",
inf_origin=-400, sup_origin=NaN, end_time=0., spec_wind_size=0.1, exti_wind_size=0.1,
foss_wind_size=0.02, spec_init=0.5, exti_init=0.5, foss_init=0.1, iter=1000, prop = 0.75,
al = 0.75, burn = 25000, thin = 200, step = 0.01)
```

This distribution can be plotted with (Fig. 1-right)

```
>plot(distSpe, type = 'l')
```

`FBDextinction_distribution` returns the list of distributions or densities of the extinction times associated to the taxa and/or the sets of taxa provided as parameters and still writes two coda files for diagnostic purposes.

```
>distExt = FBDextinction_distribution(tableTaxaName=c("Dimetrodon_natalis", "Dimetrodon_
dollovianus", "Dimetrodon_macrospondylus"), tableCladeName=("Clade_Dimetrodon.txt"),
treeName="Dimetrodon.phy", fossilName="CotylosauriaAges.csv", outName = "Dimetrodon.out",
indName = "Dimetrodon.ind", inf_origin=-400, sup_origin=NaN, end_time=0., spec_wind_size=0.1,
exti_wind_size=0.1, foss_wind_size=0.02, spec_init=0.5, exti_init=0.5, foss_init=0.1, iter=1000,
prop = 0.75, al = 0.75, burn = 25000, thin = 200, step = 0.01)
```

`FBDextinction_upperBound` returns the vector of confidence upper bounds at the order provided as parameter, of the extinction times associated to the taxa and/or the sets of taxa provided as parameters and still writes two coda files for diagnostic purposes.

```
>bound = FBDextinction_upperBound(order = 0.95, tableTaxaName=c("Dimetrodon_natalis", "Dimetrodon_
dollovianus", "Dimetrodon_macrospondylus"), tableCladeName=("Clade_Dimetrodon.txt"), treeName="Dimet
phy", fossilName="CotylosauriaAges.csv", outName = "Dimetrodon.out", indName = "Dimetrodon.
ind", inf_origin=-400, sup_origin=NaN, end_time=0., spec_wind_size=0.1, exti_wind_size=0.1,
foss_wind_size=0.02, spec_init=0.5, exti_init=0.5, foss_init=0.1, iter=1000, prop = 0.75,
al = 0.75, burn = 25000, thin = 200, order = 0.951)
```

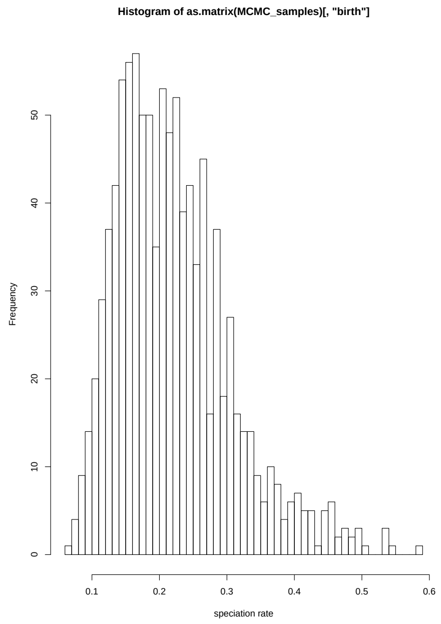

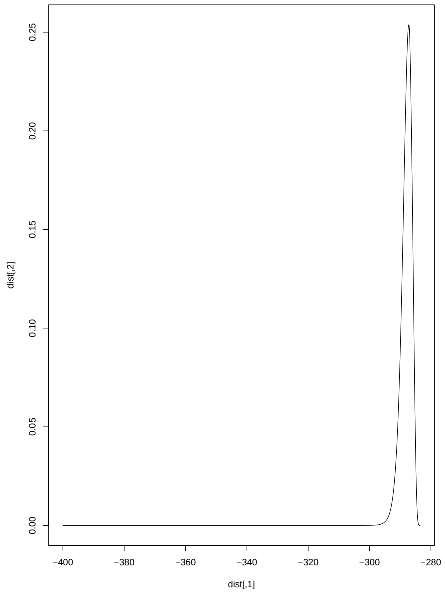

FIGURE 1 – Left. the result of the command `hist(as.matrix(MCMC_samples)[,"birth"], xlab="speciation rate", breaks=40)`. Right. the result of the command `plot(distrib, type = 'l')`

`FBDextinction_comparison` returns the the probability that a set of extinct taxa goes extinct before another and still writes two coda files for diagnostic purposes.

```
>prob = FBDextinction_comparison(tableTaxaNameA=c("Dimetrodon_natalis", "Dimetrodon_dollovianus",
"Dimetrodon_macrospondylus"), tableTaxaNameB=c("Limnoscelis_dynatis", "Limnoscelis_paludis"),
treeName="Dimetrodon.phy", fossilName="CotylosauriaAges.csv", outName = "Dimetrodon.out",
indName = "Dimetrodon.ind", inf_origin=-400, sup_origin=NaN, end_time=0., spec_wind_size=0.1,
exti_wind_size=0.1, foss_wind_size=0.02, spec_init=0.5, exti_init=0.5, foss_init=0.1, iter=1000,
prop = 0.75, al = 0.75, burn = 25000, thin = 200, order = 0.951)
```
