# Supplementary material for: Distributions of extinction times from fossil ages and tree topologies: the example of mid-Permian synapsid extinctions
Source: PeerJ. 2021 Dec 9;9:e12577. doi: 10.7717/peerj.12577 (PMC8667717; doi:10.7717/peerj.12577)
Supplement: Supplemental Information 1 — Archive of the github repository [file peerj-09-12577-s001.zip › DateFBD-master/data/Speciation/Didier & Laurin SOM 4 Biblio.docx]

Didier & Laurin SOM 4, references used to establish geological ages of the fossils.

Anderson J.S., Reisz R.R. 2004. *Pyozia mesenensis*, a new, small varanopid (Synapsida, Eupelycosauria) from Russia: "pelycosaur" diversity in the Middle Permian. J. Vertebr. Paleontol. 24:173-179.

Benson R.B.J. 2012. Interrelationships of basal synapsids: cranial and postcranial morphological partitions suggest different topologies. J. Syst. Palaeontol. 10:601–624.

Benton M.J. 1993. Reptilia. Pages 681-715 (M. J. Benton, ed.) Chapman & Hall, London.

Berman D.S. 1971. A small skull of the Lower Permian reptile *Diadectes* from the Washington formation, Dunkard group, West Virginia. Carnegie Mus. Nat. Hist. 43:33-46.

Berman D.S. 1977. A new species of *Dimetrodon* (Reptilia, Pelycosauria) from a non-deltaic facies in the Lower Permian of North-Central New Mexico. J. Paleont. 51:108-115.

Berman D.S. 1978. *Ctenospondylus ninevehensis*, a new species (Reptilia, Pelycosauria) from the Lower Permian Dunkard Group of Ohio. Carnegie Mus. Nat. Hist. 47:493-514.

Berman D.S. 1979. *Edaphosaurus* (Reptilia, Pelycosauria) from the Lower Permian of Northeastern United States, with description of a new species. Carnegie Mus. Nat. Hist. 48:185-202.

Berman D.S., Reisz R.R. 1982. Restudy of *Mycterosaurus longiceps* (Reptilia, Pelycosauria) from the Lower Permian of Texas. Carnegie Mus. Nat. Hist. 51:423–453.

Berman D.S., Reisz R.R. 1986. Captorhinid reptiles from the Early Permian of New Mexico, with description of a new genus and species. Carnegie Mus. Nat. Hist. 55:1–28.

Berman D.S., Sumida S.S. 1990. A new species of *Limnoscelis* (Amphibia, Diadectomorpha) from the Late Pennsylvanian Sangre de Cristo formation of central Colorado. Carnegie Mus. Nat. Hist. 59:303-341.

Berman D.S., Sumida S.S. 1995. New cranial material of the rare diadectid *Desmatodon hesperis* (Diadectomorpha) from the Late Pennsylvanian of Central Colorado. Carnegie Mus. Nat. Hist. 64:315-336.

Berman D.S., Henrici A.C., Sumida S.S., Martens T., Pelletier V. 2014. First European record of a varanodontine (Synapsida: Varanopidae): Member of a unique Early Permian upland paleoecosystem, Tambach Basin, central Germany. Pages 69-86 *in* Early evolutionary history of the Synapsida (C. F. Kammerer, K. D. Angielczyk, and J. Fröbisch, eds.). Springer.

Berman D.S., Reisz R.R., Bolt J.R., Scott D. 1995. The cranial anatomy and relationships of the synapsid *Varanosaurus* (Eupelycosauria: Ophiacodontidae) from the Early Permian of Texas and Oklahoma. Carnegie Mus. Nat. Hist. 64:99–133.

Berman D.S., Reisz R.R., Scott D., Henrici A.C., Sumida S.S., Martens T. 2000. Early Permian bipedal reptile. Science 290:969-972.

Berman D.S., Reisz R.R., Martens T., Henrici A.C. 2001. A new species of *Dimetrodon* (Synapsida: Sphenacodontidae) from the Lower Permian of Germany records first occurrence of genus outside of North America. Can. J. Earth Sci. 38:803–812.

Berman D.S., Sumida S.S., Martens T. 1998. *Diadectes* (Diadectomorpha: Diadectidae) from the Early Permian of central Germany, with description of a new species. Carnegie Mus. Nat. Hist. 67:53-93.

Berman D.S., Henrici A.C., Kissel R.A., Sumida S.S., Martens T. 2004. A new diadectid (Diadectomorpha), *Orobates pabsti*, from the early Permian of Central Germany. Bulletin of Carnegie Museum of natural History 35:1–36.

Botha-Brink J., Modesto S.P. 2007. A mixed-age classes 'pelycosaur' aggregation from South Africa: earliest evidence of parental care in amniotes? Proc. R. Soc. Lond. B 274:2829–2834.

Botha-Brink J., Modesto S.P. 2009. Anatomy and relationships of the Middle Permian Varanopid *Heleosaurus scholtzi* based on a social aggregation from the Karoo Basin of South Africa. J. Vertebr. Paleontol. 29:389–400.

Boy J.A., Martens T. 1991. Ein neues captorhinomorphes Reptil aus dem thüringischen Rotliegend (Unter-Perm; Ost-Deutschland). Paläontol. Z. 65:363-389.

Brink K.S., Maddin H.C., Evans D.C., Reisz R.R., Sues H.-D. 2015. Re-evaluation of the historic Canadian fossil *Bathygnathus borealis* from the Early Permian of Prince Edward Island. Can. J. Earth Sci. 52:1109-1120.

Brinkman D., Eberth D.A. 1986. The anatomy and relationships of Stereophallodon and Baldwinonus (Reptilia, Pelycosauria). Breviora 485:1-34.

Brocklehurst N. 2017. Rates of morphological evolution in Captorhinidae: An adaptive radiation of Permian herbivores. PeerJ 5:e3200.

Brocklehurst N., Fröbisch J. 2017. A re-examination of the enigmatic Russian tetrapod *Phreatophasma aenigmaticum* and its evolutionary implications. Foss. Rec. 20:87–93.

Brocklehurst N., Fröbisch J. 2018. A reexamination of *Milosaurus mccordi*, and the evolution of large body size in Carboniferous synapsids. J. Vertebr. Paleontol. 35:1-10.

Brocklehurst N., Reisz R.R., Fernandez V., Fröbisch J. 2016. A Re-Description of *‘Mycterosaurus’ smithae,* an Early Permian Eothyridid, and Its Impact on the Phylogeny of Pelycosaurian-Grade Synapsids. PLoS ONE 11:e0156810.

Campione N.s.E., Reisz R.R. 2010. *Varanops brevirostris* (Eupelycosauria: Varanopidae) from the Lower Permian of Texas, with discussion of varanopid morphology and interrelationships. J. Vertebr. Paleontol. 30:724–746.

Carpenter D.K., Falcon‐Lang H.J., Benton M.J., Grey M. 2015. Early Pennsylvanian (Langsettian) fish assemblages from the Joggins Formation, Canada, and their implications for palaeoecology and palaeogeography. Palaeontology 58:661–690.

Carroll R.L. 1969. A Middle Pennsylvanian captorhinomorph, and the interrelationships of primitive reptiles. J. Paleont. 43:151–170.

Carroll R.L., Baird D. 1972. Carboniferous Stem-Reptiles of the Family Romeriidaed. Bull. Mus. comp. Zool. Harv. 143:321-364.

Case E.C. 1907. A revision of the Pelycosauria of North America. Carnegie Institution of Washington, Publication 55:1-176.

Case E.C. 1910. New or little known reptiles and amphibians from the Permian (?) of Texas. Bull. Am. Mus. Nat. Hist. 28:163-181.

Case E.C. 1911. A revision of the Cotylosauria of North America. Carnegie Inst. Washington 145:1-122.

Case E.C., Williston S.W., Mehl M.G. 1913. Permo-Carboniferous vertebrates from New Mexico. Publ. Carnegie Inst. Washington 181:1–81.

Clark J., Carroll R.L. 1973. Romeriid Reptiles from the Lower Permian. Bull. Mus. comp. Zool. Harv. 144:353-407.

Cisneros J.C., Rubidge B.S., Mason R., Dube C. 2008. Analysis of millerettid parareptile relationships in the light of new material of *Broomia perplexa* Watson, 1914, from the Permian of South Africa. J. Syst. Palaeontol. 6:453–462.

Cope E.D. 1875. On fossil remains of reptilia and fishes from Illinois. Proc. Acad. Nat. Sci. Phila. 27:404-408.

Cope E.D. 1878. Descriptions of extinct Batrachia and Reptilia from the Permian formation of Texas. Proc. Am. Phil. Soc. 17:505–530.

Cope E.D. 1882. Third contribution to the history of the Vertebrata of the Permian formation of Texas. Proc. Am. Phil. Soc. 20:447-461.

Cope E.D. 1885. A contribution ot the vertebrate paleontology of Brazil. Paleontol. Bull. 40:1–21.

Cope E.D. 1896. Second contribution to the history of the Cotylosauria. Proc. Am. Phil. Soc.:122-139.

Currie P.J. 1977. A new haptodontine sphenacodont (Reptilia: Pelycosauria) from the Upper Pennsylvanian of North America. J. Paleont. 51:927-942.

Day M.O., Güven S., Abdala F., Jirah S., Rubidge B., Almond J. 2015. Youngest dinocephalian fossils extend the Tapinocephalus Zone, Karoo Basin, South Africa. S. Afr. J. Sci. 111:1-5.

deBraga M., Reisz R.R. 1995. A new diapsid reptile from the uppermost Carboniferous (Stephanian) of Kansas. Palaeontology 38:199-212.

deBraga M., Reisz R.R. 1996. The Early Permian reptile *Acleistorhinus pteroticus* and its phylogenetic position. J. Vertebr. Paleontol. 16:384–395.

Dilkes D.W., Reisz R.R. 1996. First record of a basal synapsid ('mammal-like reptile') in Gondwana. Proc. R. Soc. Lond. B 263:1165-1170.

DiMichele W.A., Tabor N.J., Chaney D.S. 2005. Outcrop-scale environmentla heterogeneity and vegetational complexity in the Permo-Carboniferous Markley formation of north-central Texas. NMMNH Bulletin 30, The Nonmarine Permian:60–66.

Dodick J.T., Modesto S.P. 1995. The cranial anatomy of the captorhinid reptile *Labidosaurikos meachami* from the Lower Permian of Oklahoma. Palaeontology 38:687-711.

Eberth D.A. 1985. The skull of *Sphenacodon ferocior*, and comparisons with other sphenacodontines (Reptilia: Pelycosauria). New Mexico Bureau of Mines and Mineral Resources Circular 190:1-39.

Eberth D.A., Brinkman D. 1983. *Ruthiromia elcobriensis*, a new pelycosaur from El Cobre Canyon, New Mexico. Breviora 474:1-26.

Efremov J.A. 1938. Some new Permian reptiles of the USSR. C. R. (Doklady) Acad. Sci. URSS 19:771-776.

Florides G.A., Kalogirou S.A., Tassou S.A., Wrobel L. 2001. Natural environment and thermal behaviour of *Dimetrodon limbatus*. J. Therm. Biol. 26:15-20.

Fracasso M.A. 1983. Cranial osteology, functional morphology, systematics and paleoenvironment of *Limnoscelis paludis* Williston. Yale, New Haven, Connecticut, 624 pp.

Fröbisch J., Schoch R.R., Müller J., Schindler T., Schweiss D. 2011. A new basal sphenacodontid synapsid from the Late Carboniferous of the Saar−Nahe Basin, Germany. Acta Palaeont. Pol. 56:113–120.

Gaudry A. 1880. Sur un reptile très perfectionné trouvé dans le terrain permien. C. R. Acad. Sci. Paris 91:669–671.

Gradstein F.M., Ogg J.G., Smith A.G. (eds) 2004. A Geologic Time Scale 2004. Cambridge University Press, Cambridge.

Heaton M.J. 1979. Cranial anatomy of primitive captorhinid reptiles from the Late Pennsylvanian and Early Permian Oklahoma and Texas. Bull. Okla. geol. Surv. 127:1-84.

Heaton M.J., Reisz R.R. 1980. A skeletal reconstruction of the Early Permian captorhinid reptile *Eocaptorhinus laticeps* (Williston). J. Paleont. 54:136-143.

Hentz T.F. 1988. Lithostratigraphy and paleoenvironments of Upper Paleozoic Continental Red Beds, North-Central Texas: Bowie (New) and Wichita (Revised) Groups. Rep. Invest. Bur. econ. Geol. Univ. Tex. 170:1-55.

Holmes R.B., Carroll R.L., Reisz R.R. 1998. The first articulated skeleton of *Dendrerpeton acadianum* (Temnospondyli, Dendrerpetontidae) from the Lower Pennsylvanian locality of Joggins, Nova Scotia, and a review of its relationships. J. Vertebr. Paleontol. 18:64-79.

Hook R.W., Hotton N., III. 1991. A new sphenacodontid pelycosaur (Synapsida) from the Wichita Group, Lower Permian of north-central Texas. J. Vertebr. Paleontol. 11:37–44.

Ivakhnenko M.F., Kurzanov S.M. 1978. *Mesenosaurus*, a primitive archosaur. Paleont. Jour. 1978:139-141.

Kennedy N.K. 2010. Redescription of the postcranial skeleton of *Limnoscelis paludis* Williston (Diadectomorpha: Limnoscelidae) from the Upper Pennsylvanian of El Cobre Cañon, Northern New Mexico. New Mexico Mus. Nat. Hist. Sci. Bull. 49:211–220.

Kissel R. 2010. Morphology, Phylogeny, and Evolution of Diadectidae (Cotylosauria: Diadectomorpha). University of Toronto, Toronto, xv + 185 pp.

Kissel R.A., Lehman T.M. 2002. Upper Pennsylvanian tetrapods from the Ada Formation of Seminole County, Oklahoma. J. Paleont. 76:529–545.

Kissel R.A., Reisz R.R. 2004a. *Ambedus pusillus*, new genus, new species, a small diadectid (Tetrapoda: Diadectomorpha) from the Lower Permian of Ohio with a consideration of diadectomorph phylogeny. Annals of Carnegie Museum 73:197-212.

Kissel R.A., Reisz R.R. 2004b. Synapsid fauna of the Upper Pennsylvanian Rock Lake Shale near Garnett, Kansas and the diversity pattern of early amniotes. Pages 409-428 (G. Arratia, M. V. H. Wilson, and R. Cloutier, eds.). Verlag Dr. Friedrich Pfeif, München.

Kissel R.A., Dilkes D.W., Reisz R.R. 2002. *Captorhinus magnus*, a new captorhinid (Amniota: Eureptilia) from the Lower Permian of Oklahoma, with new evidence on the homology of the astragalus. Can. J. Earth Sci. 39:1363-1372.

Langston W. 1965. *Oedaleops campi* (Reptilia: Pelycosauria) new genus and species from the Lower Permian of New Mexico, and the family Eothyrididae. Bull. Texas Mem. Mus. 9:1-47.

Langston W., Reisz R.R. 1981. *Aerosaurus wellesi*, new species, a varanopseid mammal-like reptile (Synapsida: Pelycosauria) from the Lower Permian of New Mexico. J. Vertebr. Paleontol. 1:73-96.

Laurin M. 1993. Anatomy and relationships of *Haptodus garnettensis*, a Pennsylvanian synapsid from Kansas. J. Vertebr. Paleontol. 13:200–229.

Laurin M. 1994. Re-evaluation of *Cutleria wilmarthi,* an Early Permian synapsid from Colorado. J. Vertebr. Paleontol. 14:134-138.

Lewis G.E., Vaughn P.P. 1965. Early Permian Vertebrates from the Cutler Formation of the Placerville Area Colorado. Prof. Pap. U.S. geol. Surv. 503-C:1-50.

Liu J., Rubidge B., Li J. 2010. A new specimen of *Biseridens qilianicus* indicates its phylogenetic position as the most basal anomodont. Proc. R. Soc. Lond. B 277:285–292.

Lucas S.G. 2006. Global Permian tetrapod biostratigraphy and biochronology. Pages 65–93 (S. G. Lucas, G. Cassinis, and J. W. Schneider, eds.). The Geological Society of London, London.

Lucas S.G., Harris S.K., Spielmann J.A., Rinehart L.F., Berman D.S., Henrici A.C., Krainer K. 2010. Vertebrate paleontology, biostratigraphy and biochronology of the Pennsylvanian-Permian Cutler group, Cañon del Cobre, northern New Mexico. New Mexico Mus. Nat. Hist. Sci. Bull. 49:115–124.

Lucas S.G., Rinehart L.F., Celeskey M.D. 2018. The oldest specialized tetrapod herbivore: A new eupelycosaur from the Permian of New Mexico, USA. Palaeontol. Electron. 21:1-42.

Lyson T.R., Bever G.S., Bhullar B.-A.S., Joyce W.G., Gauthier J.A. 2010. Transitional fossils and the origin of turtles. Biol. Lett. 6:830–833.

MacLean 3rd W.P. 1970. The braincase of *Labidosaurikos* (a Permian captorhinomorph reptile). J. Paleont.:458-463.

Maddin H.C., Evans D.C., Reisz R.R. 2006. An Early Permian varanodontine varanopid (Synapsida: Eupelycosauria) from the Richards Spur locality, Oklahoma. J. Vert. Paleont. 26:957–966.

Maddin H.C., Sidor C.A., Reisz R.R. 2008. Cranial anatomy of *Ennatosaurus tecton* (Synapsida: Caseidae) from the Middle Permian of Russia and the evolutionary relashionships of Caseidae. J. Vertebr. Paleontol. 28:160–180.

Marsh O.C. 1878. Notice of new fossil reptiles. Am. Jour. Sci.—Third Series 15:409-411.

Mazierski D.M., Reisz R.R. 2010. Description of a new specimen of *Ianthasaurus hardestiorum* (Eupelycosauria: Edaphosauridae) and a re-evaluation of edaphosaurid phylogeny. Can. J. Earth Sci. 47:901–912.

Modesto S.P. 1994. The Lower Permian synapsid *Glaucosaurus* from Texas. Palaeontology 37:51–60.

Modesto S.P. 1995. The skull of the herbivorous synapsid *Edaphosaurus boanerges* from the Lower Permian of Texas. Palaeontology 38:213-239.

Modesto S. 2006. The cranial skeleton of the Early Permian aquatic reptile *Mesosaurus tenuidens*: implications for relationships and palaeobiology. Zool. J. Linn. Soc. 146:345-368.

Modesto S.P. 2010. The postcranial skeleton of the aquatic parareptile *Mesosaurus tenuidens* from the Gondwanan Permian. J. Vertebr. Paleontol. 30:1378–1395.

Modesto S.P., Reisz R.R. 1990. A new skeleton of *Ianthasaurus hardestii*, a primitive edaphosaur (Synapsida: Pelycosauria) from the Late Pennsylvanian of Kansas. Can. J. Earth Sci. 27:834–844.

Modesto S.P., Reisz R.R. 1992. Restudy of Permo-Carboniferous synapsid *Edaphosaurus novomexicanus* Williston and Case, the oldest known herbivorous amniote. Can. J. Earth Sci. 29:2653–2662.

Modesto S., Smith R.M.H. 2001. A new Late Permian captorhinid reptile: a first record from the South African Karoo. J. Vertebr. Paleontol. 21:405-409.

Modesto S.P., Lamb A.J., Reisz R.R. 2014. The captorhinid reptile *Captorhinikos valensis* from the lower Permian Vale Formation of Texas, and the evolution of herbivory in eureptiles. J. Vertebr. Paleontol. 34:291–302.

Modesto S., Sidor C.A., Rubidge B.S., Welman J. 2001. A second varanopseid skull from the Upper Permian of South Africa: implications for Late Permian 'pelycosaur' evolution. Lethaia 34:249-259.

Moss J.L. 1972. The morphology and phylogenetic relationships of the lower Permian tetrapod *Tseajaia campi* Vaughn (Amphibia: Seymouriamorpha). Univ. Calif. Publs Bull. Dep. Geol. 98:1-71.

Müller J., Reisz R.R. 2005. An early captorhinid reptile (Amniota, Eureptilia) from the Upper Carboniferous of Hamilton, Kansas. J. Vertebr. Paleontol. 25:561–568.

Müller J., Li J.-L., Reisz R.R. 2008. A new bolosaurid parareptile, *Belebey chengi* sp. nov., from the Middle Permian of China and its paleogeographic significance. Naturwissenschaften 95:1169–1174.

Müller J., Berman D.S., Henrici A.C., Martens T., Sumida S.S. 2006. The basal reptile *Thuringothyris mahlendorffae* (Amniota: Eureptilia) from the Lower Permian of Germany. J. Paleont. 80:726–739.

Ogg J.G., Ogg G., Gradstein F.M. 2016. A concise geologic time scale: 2016. Elsevier, Amsterdam.

Olson E.C. 1947. The family Diadectidae and its bearing on the classification of reptiles. Fieldiana, Geol. 11:1-53.

Olson E.C. 1954. Fauna of the Vale and Choza: 7—Pelycosauria: Family Caseidae. Fieldiana, Geol. 10:193-204.

Olson E.C. 1962. Permian Vertebrates Oklahoma and Texas. Part 2.—The Osteology of *Captorhinikos chozaensis* Olson. Circ. Okla. geol. Surv. 59:49-68.

Olson E.C. 1965. New Permian Vertebrates from the Chickasha Formation in Oklahoma. Circ. Okla. geol. Surv. 70:1-70.

Olson E.C. 1968. The family Caseidae. Fieldiana, Geol. 17:225-349.

Olson E.C. 1970. New and little known genera and species of vertebrates from the Lower Permian of Oklahoma. Fieldiana, Geol. 18:359-434.

Olson E.C. 1984. The taxonomic status and morphology of *Pleuristion brachycoelus* Case; referred to *Protocaptorhinus price*i Clark and Carroll (Reptilia: Captorhinimorpha). J. Paleont. 58:1282–1295.

Olson E.C., Barghusen H. 1962. Permian Vertebrates Oklahoma and Texas. Part 1.—Vertebrates from the Flowerpot Formation, Permian of Oklahoma. Circ. Okla. geol. Surv. 59:5-48.

Olson E.C., Beerbower J.R. 1953. The San Angelo Formation, Permian of Texas, and its vertebrates. J. Geol. (Chicago) 61:389–423.

Paton R.L. 1974. Lower Permian pelycosaurs from the English Midlands. Palaeontology 17:541–552.

Reisz R.R. 1972. Pelycosaurian reptiles from the Middle Pennsylvanian of North America. Bull. Mus. comp. Zool. Harv. 144:27-62.

Reisz R.R. 1986. Pelycosauria. Pages 1–102 *in* Encyclopedia of Paleoherpetology (P. Wellnhofer, ed.) Gustav Fischer, Stuttgart.

Reisz R.R. 2005. *Oromycter*, a new caseid from the Lower Permian of Oklahoma. J. Vertebr. Paleontol. 25:905-910.

Reisz R.R., Baird D. 1983. Captorhinomorph “stem” reptiles from the Pennsylvanian coal-swamp deposit of Linton, Ohio. Carnegie Mus. Nat. Hist. 52:393–411.

Reisz R.R., Berman D.S. 1986. *Ianthasaurus hardestii* n. sp., a primitive edaphosaur (Reptilia, Pelycosauria) from the Upper Pennsylvanian Rock Lake Shale near Garnett, Kansas. Can. J. Earth Sci. 23:77–91.

Reisz R.R., Berman D.S. 2001. The skull of *Mesenosaurus romeri,* a small varanopseid (Synapsida: Eupelycosauria) from the Upper Permian of the Mezen river basin, northern Russia. Carnegie Mus. Nat. Hist. 70:113-132.

Reisz R.R., Dilkes D.W. 1992. The taxonomic position of *Anningia megalops*, a small amniote from the Permian of South Africa. Can. J. Earth Sci. 29:1605–1608.

Reisz R.R., Dilkes D.W. 2003. *Archaeovenator hamiltonensis*, a new varanopid (Synapsida: Eupelycosauria) from the Upper Carboniferous of Kansas. Can. J. Earth Sci. 40:667-678.

Reisz R.R., Fröbisch J. 2014. The oldest caseid synapsid from the Late Pennsylvanian of Kansas, and the evolution of herbivory in terrestrial vertebrates. PLoS ONE 9:1–9.

Reisz R.R., Laurin M. 2004. A reevaluation of the enigmatic Permian synapsid *Watongia* and of its stratigraphic significance. Can. J. Earth Sci. 41:377-386.

Reisz R.R., Modesto S.P. 2007. *Heleosaurus scholtzi* from the Permian of South Africa: a varanopid synapsid, not a diapsid reptile. J. Vertebr. Paleontol. 27:727-733.

Reisz R.R., Tsuji L.A. 2006. An articulated skeleton of *Varanops* with bite marks: the oldest known evidence of scavenging among terrestrial vertebrates. J. Vert. Paleont. 26:1021–1023.

Reisz R.R., Barkas V., Scott D. 2002. A new early permian Bolosaurid reptile from the Richards Spur Dolese Brothers Quarry, near fort sill, Oklahoma. J. Vertebr. Paleontol. 22:23-28.

Reisz R.R., Berman D.S., Scott D. 1992. The cranial anatomy and relationships of *Secodontosaurus*, an unusual mammal-like reptile (Synapsida: Sphenacodontidae) from the early Permian of Texas. Zool. J. Linn. Soc. 104:127–184.

Reisz R.R., Dilkes D.W., Berman D.S. 1998. Anatomy and relationships of *Elliotsmithia longiceps* Broom, a small synapsid (Eupelycosauria: Varanopseidae) from the Late Permian of South Africa. J. Vertebr. Paleontol. 18:602-611.

Reisz R.R., Godfrey S., Scott D. 2009. *Eothyris* and *Oedaleops*: Do these early Permian synapsids form a clade? J. Vertebr. Paleontol. 29:39–47.

Reisz R.R., Liu J., Li J.-L., Müller J. 2011. A new captorhinid reptile, *Gansurhinus qingtoushanensis*, gen. et sp. nov., from the Permian of China. Naturwissenschaften 98:435–441.

Reisz R.R., Laurin M., Marjanović D. 2010. *Apsisaurus witteri* from the Lower Permian of Texas: yet another small varanopid synapsid, not a diapsid. J. Vertebr. Paleontol. 30:1628–1631.

Reisz R.R., Maddin H.C., Fröbisch J., Falconnet J. 2011. A new large caseid (Synapsida, Caseasauria) from the Permian of Rodez (France), including a reappraisal of *“Casea” rutena* Sigogneau-Russell & Russell, 1974. Geodiversitas 33:227–246.

de Ricqlès A., Taquet P. 1982. La faune de vertébrés du Permien Supérieur du Niger I. Le captorhinomorphe *Moradisaurus grandis* (Reptilia, Cotylosauria)—le crâne—. Annls. Paléont. 68:33-106.

Romano M., Ronchi A., Maganuco S., Nicosia U. 2017. New material of *Alierasaurus ronchii* (Synapsida, Caseidae) from the Permian of Sardinia (Italy), and its phylogenetic affinities. Palaeontol. Electron. 20:1-27.

Romer A.S. 1925. An ophiacodont reptile from the Permian of Kansas. J. Geol. (Chicago) 33:173-182.

Romer A.S. 1927. Notes on the Permo-Carboniferous reptile *Dimetrodon*. J. Geol. (Chicago) 35:673-689.

Romer A.S. 1937. New genera and species of pelycosaurian reptiles. New England Zool. Club Pr. 16:89-96.

Romer A.S. 1952. Late Pennsylvanian and Early-Permian vertebrates of the Pittsburgh-West Virginia. Carnegie Mus. Nat. Hst. 33:47–113.

Romer A.S., Price L.I. 1940. Review of the Pelycosauria. Arno Press, New York.

Ronchi A., Sacchi E., Romano M., Nicosia U. 2011. A huge caseid pelycosaur from north−western Sardinia and its bearing on European Permian stratigraphy and palaeobiogeography. Acta Palaeont. Pol. 56:723–738.

Rubidge B.S., Modesto S., Sidor C., Welman J. 1999. *Eunotosaurus africanus* from the Ecca-Beaufort contact in Northern Cape Province, South Africa—implications for Karoo Basin development. S. Afr. J. Sci. 95:553-556.

Rubidge B. 2013. The roots of early mammals lie in the Karoo: Robert Broom's foundation and subsequent research progress. Trans. Roy. Soc. S. Afr.

Ruta M., Cisneros J.C., Liebrecht T., Tsuji L.A., Müller J. 2011. Amniotes through major biological crises: faunal turnover among Parareptiles and the end-Permian mass extinction. Palaeontology 54:1117–1137.

Schneider J.W., Lucas S.G., Barrick J.E. 2013. The Early Permian age of the Dunkard Group, Appalachian basin, U.S.A., based on spiloblattinid insect biostratigraphy. Int. J. Coal Geol. 119:88–92.

Seltin R.J. 1959. A review of the family Captorhinidae. Fieldiana, Geol. 10:461–509.

Shelton C.D., Sander P.M., Stein K., Winkelhorst H. 2013. Long bone histology indicates sympatric species of *Dimetrodon* (Lower Permian, Sphenacodontidae). Earth Environ. Sci. Trans. R. Soc. Edin. 103:1–20.

Shen S.-Z., Schneider J.W., Angiolini L., Henderson C.M. 2013. The International Permian Timescale: March 2013 update. New Mexico Mus. Nat. Hist. Sci. Bull. 60:411–416.

Shikama T. 1970. On some *Mesosaurus* skeletons kept in Japan. Kamkura 16:29–49.

Shikama T., Ozaki H. 1966. On a reptiilan skeleton from the Paleozoic formation of San Paulo, Brazil. Trans. Proc. Paleont. Soc. Japan. N. S. 64:351-358.

Sigogneau-Russell D., Russell D.E. 1974. Étude du premier caséidé (Reptilia, Pelycosauria) d'Europe occidentale. Bull. Mus. natl. Hist. nat., Paris, 3è sér. 38:145-215.

Smith R.M.H., Rubidge B.S., Sidor C.A. 2006. A new burnetiid (Therapsida: Biarmosuchia) from the Upper Permian of South Africa and its biogeographic implications. J. Vertebr. Paleontol. 26:331-343.

Spindler F., Werneburg R., Schneider J.W., Luthardt L., Annacker V., Rößler R. 2017. First arboreal 'pelycosaurs' (Synapsida: Varanopidae) from the early Permian Chemnitz Fossil Lagerstätte, SE Germany, with a review of varanopid phylogeny. PalZ 92:315–364.

Stovall J.W. 1937. *Cotylorhynchus romeri*, a new genus and species of pelycosaurian reptile from Oklahoma. Am. Jour. Sci. 34:308-313.

Stovall J.W. 1950. A new cotylosaur from North Central Oklahoma. Am. Jour. Sci. 248:46-54.

Sullivan C., Reisz R.R. 1999. First record of *Seymouria* (Vertebrata: Seymouriamorpha) from Early Permian fissure fills at Richards Spur, Oklahoma. Can. J. Earth Sci. 36:1257-1266.

Sumida S.S. 1989. New information on the pectoral girdle and vertebral column in *Lupeosaurus* (Reptilia, Pelycosauria). Can. J. Earth Sci. 26:1343-1349.

Sumida S.S., Dodick J., Metcalf A., Albright G. 2010. *Reiszorhinus olsoni*, a new single-tooth-rowed captorhinid reptile from the Lower Permian of Texas. J. Vertebr. Paleontol. 30:704–714.

Taquet P. 1969. Première découverte en Afrique d'un reptile captorhinomorphe (cotylosaurien). C. R. Acad. Sci. Paris 268:779-781.

Tsuji L.A., Müller J., Reisz R.R. 2010. *Microleter mckinzieorum* gen. et sp. nov. from the Lower Permian of Oklahoma: the basalmost parareptile from Laurasia. J. Syst. Palaeontol. 8:245–255.

Vaughn P.P. 1958. On a new pelycosaur from the Lower Permian of Oklahoma, and on the origin of the family Caseidae. J. Paleont. 32:981-991.

Vaughn P.P. 1964. Vertebrates from the Organ Rock Shale of the Cutler group, Permian of Monument Valley and vicinity, Utah and Arizona. J. Paleont. 38:567-583.

Vaughn P.P. 1969. Lower Permian Vertebrates of the Four Corners and the Midcontinent as Indices of Climatic Differences *in* Proceedings of the North American Paleontological Congress. 1969:388-408.

Vaughn P.P. 1972. More vertebrates, including a new microsaur, from the Upper Pennsylvanian of central Colorado. Contr. Sci. 223:1-30.

von Huene F. 1925. Ein neuer Pelycosaurier aus der unteren Permformation Sachsens. G. Pal. Abh. 18 (Neu Folge 14):215-264.

Watson D.M.S. 1914. *Broomia perplexa,* gen. et sp. n., a fossil reptile from South Africa. J. Zool. (Lond.) 1914:995-1010.

Watson D.M.S. 1957. On *Millerosaurus* and the early history of the sauropsid reptiles. Phil. Trans. R. Soc. B 240:325-400.

Williston S. 1909. New or Little Known Permian Vertebrates. *Pariotichus*. 17:241-255.

Williston S.W. 1910. The skull of Labidosaurus. Amer. J. Anat. 10:69-84.

Williston S.W. 1911. A new family of reptiles from the Permian of New Mexico. Am. Jour. Sci. 31:378-398.

Williston S.W. 1914. The osteology of some american Permian vertebrates. J. Geol. 22:364–419.

Williston S.W. 1915. A new genus and species of American Theromorpha: *Mycterosaurus longiceps*. J. Geol. (Chicago) 23:554–559.
